# Supplementary material for: Impact of Ferrous Sulfate on Thylakoidal Multiprotein Complexes, Metabolism and Defence of Brassica juncea L. under Arsenic Stress
Source: Plants (Basel). 2022 Jun 13;11(12):1559. doi: 10.3390/plants11121559 (PMC9228442; doi:10.3390/plants11121559)
Supplement: Supplementary file 1 [file plants-11-01559-s001.zip › plants-1730660-supplementary.pdf]

$FeSO_4$: 2 mM (After 3 days of age in 1 set of 2)

As stress exposure at the age of 40 days (Both sets) for 7 and 14 days.

As concentration:
$Na_2HAsO_4.7H_2O$: 250 µM

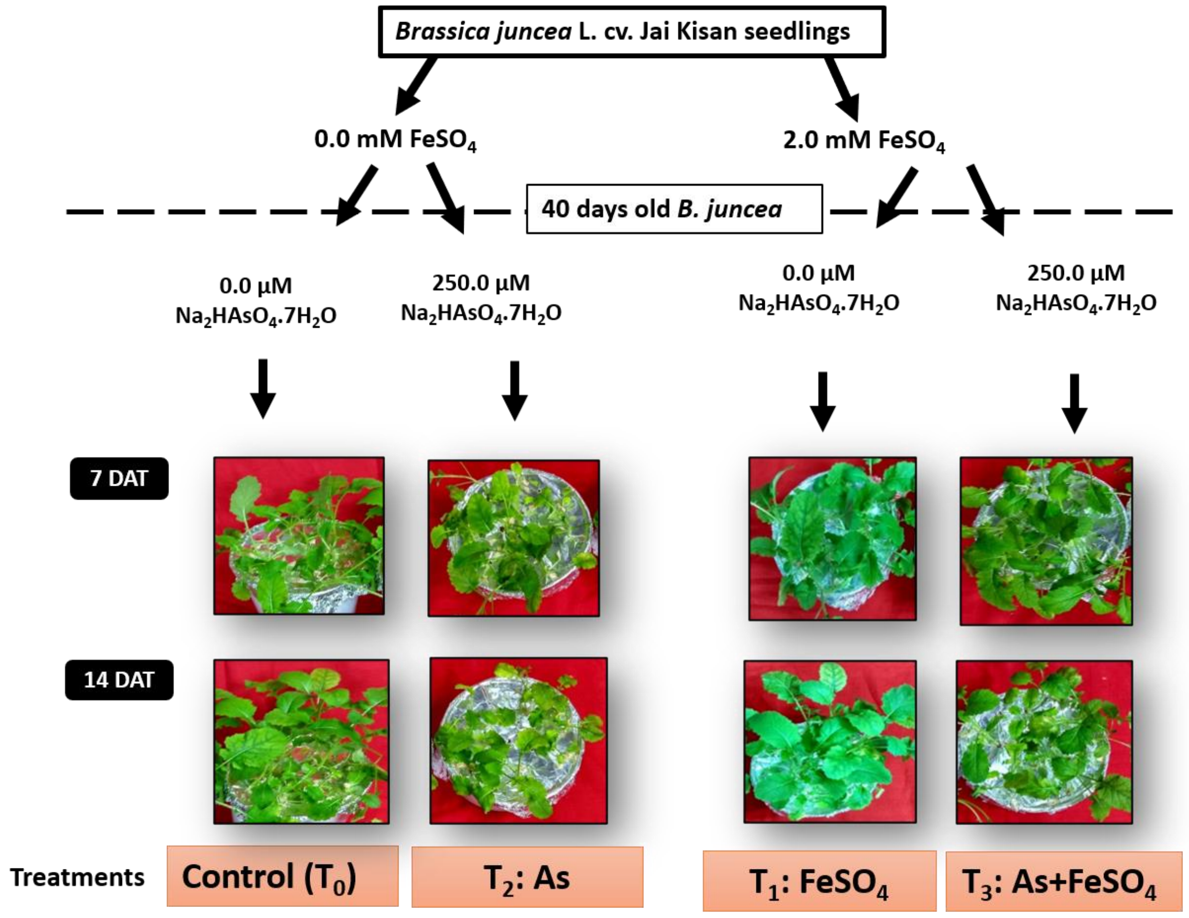

Supplementary Figure S1: Experimental design and arsenic treatments to 40-days-old *Brassica. juncea* L. plants.

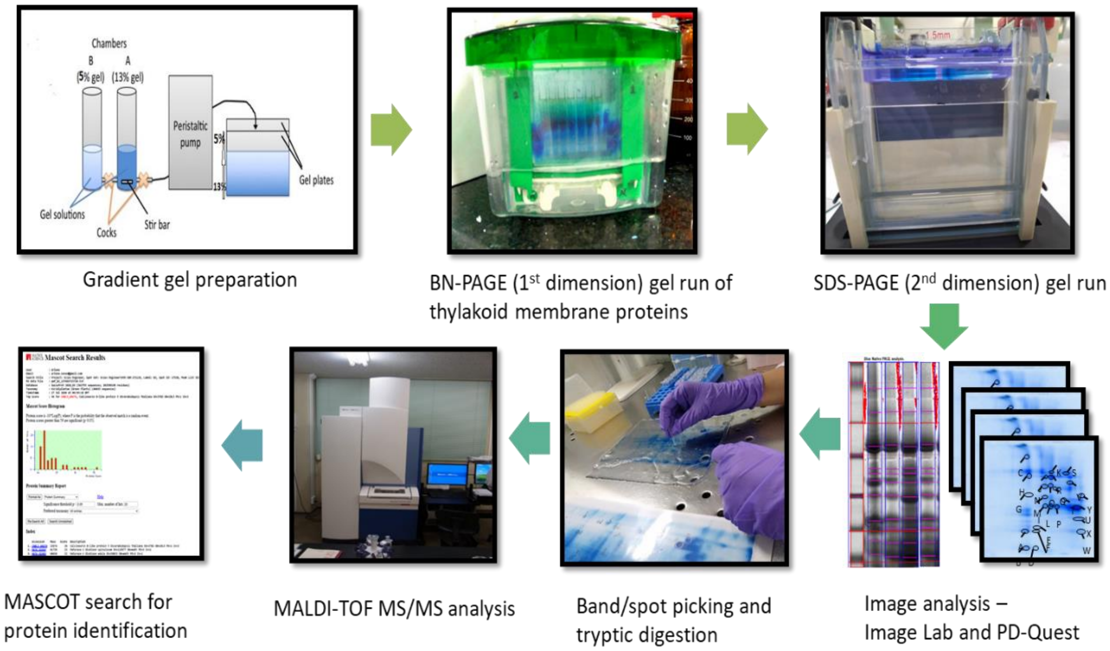

Supplementary Figure S2: An overview of proteomics methodology adopted for the present study **.**

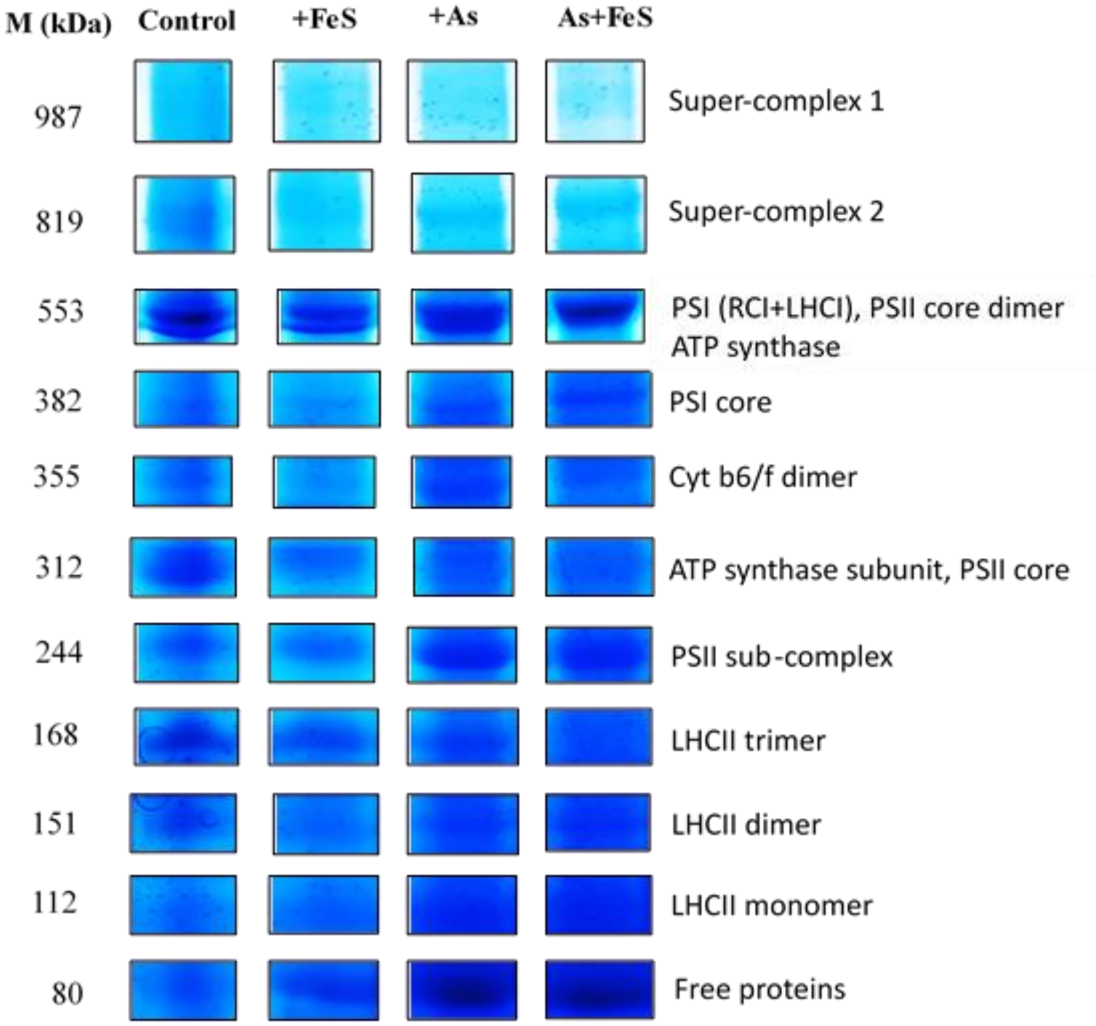

Supplementary Figure S3: Individual band profiles of thylakoidal multi-protein-pigment complexes isolated from chloroplasts of *Brassica juncea* showing the effect of arsenic (As) and /or iron-sulfate ($FeSO_4$) treatments at 14 DAT. $FeSO_4$, FeS.

A B

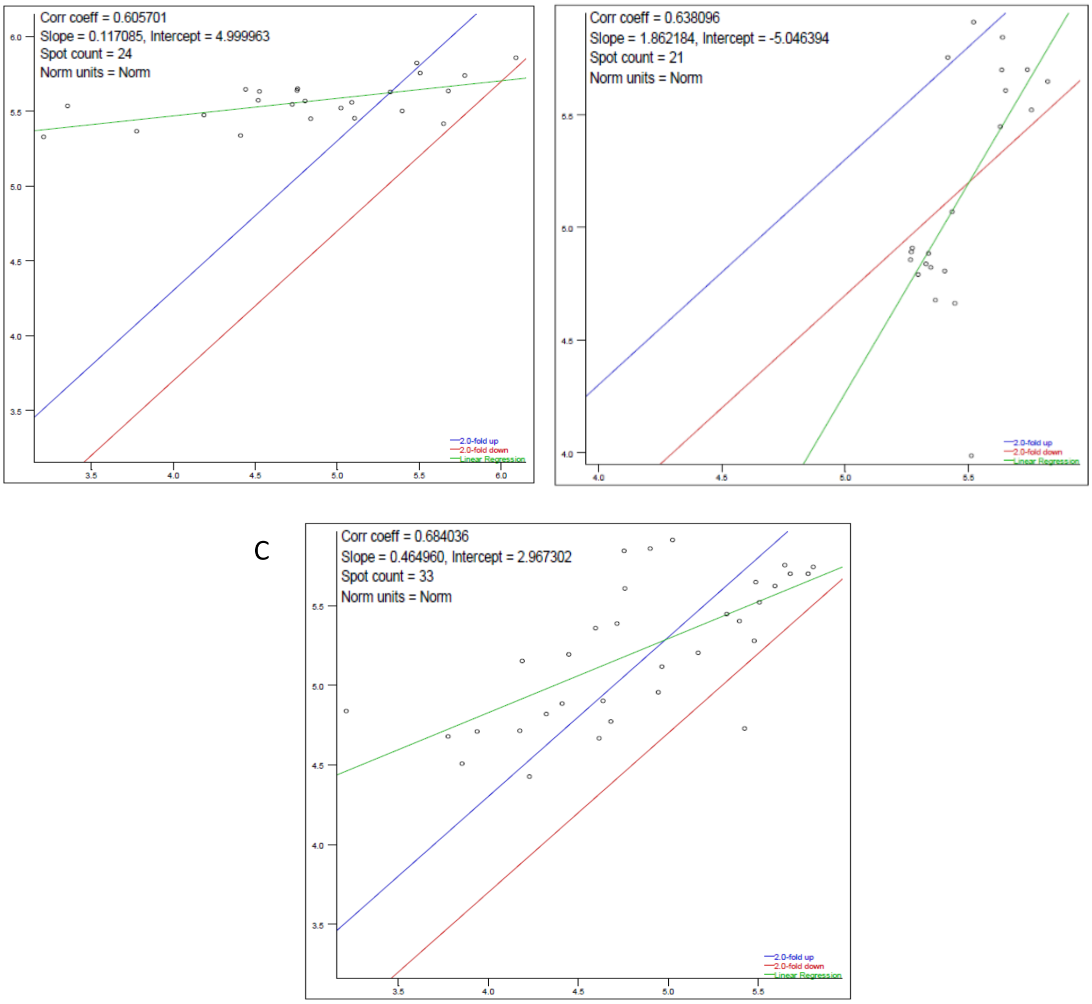

C

Supplementary Figure S4: Scatter plots depicting differential expression of thylakoidal MPC subunits in *B. juncea* L. exposed to As stress and $FeSO_4$ supplementation at 14 DAT. **A**. Control Vs (+$FeSO_4$), **B**. Control Vs (+As), **C.** Control Vs (As+$FeSO_4$). Arsenic, As.

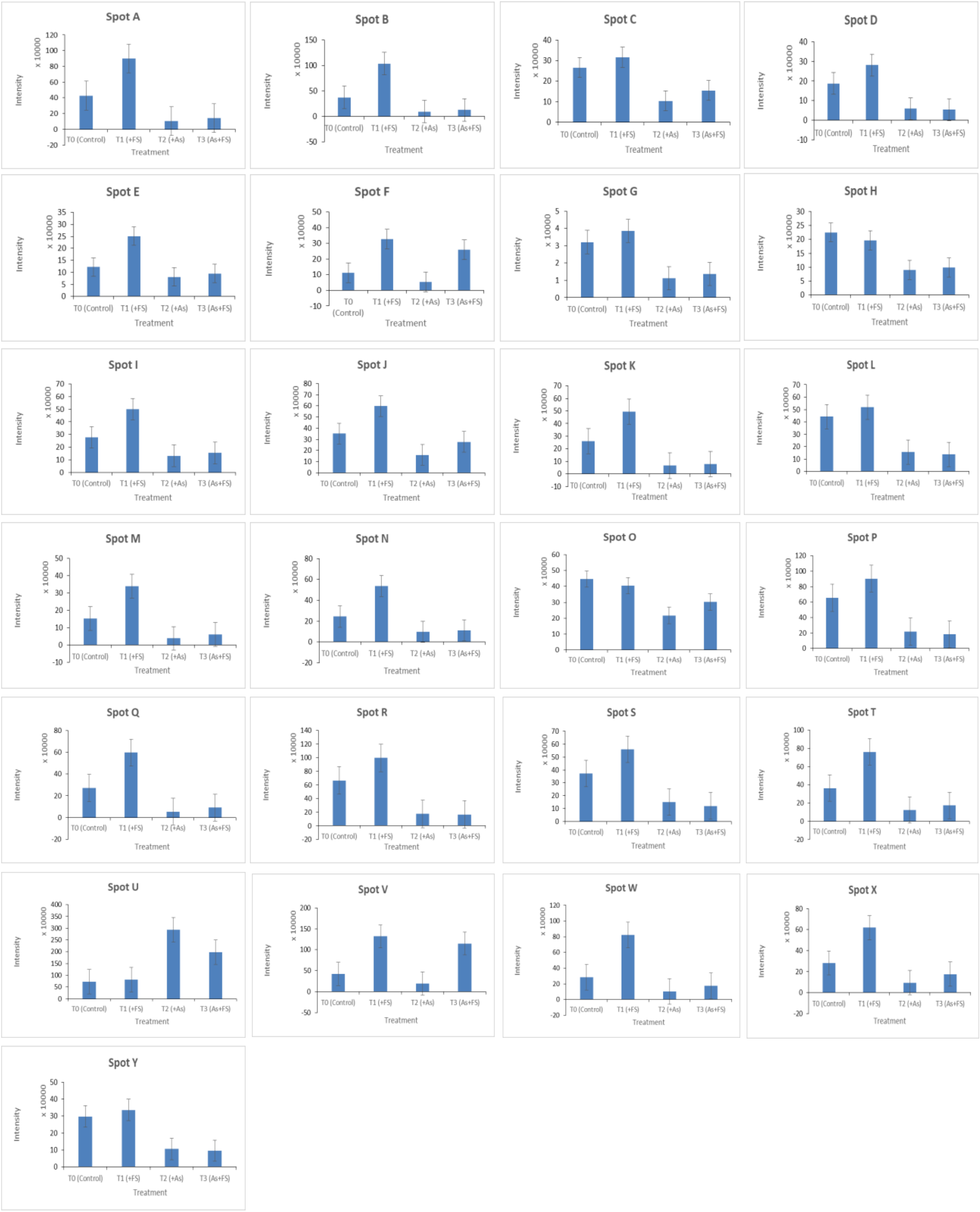

Supplementary Figure S5: Graphical representation of the differential expression of 25 spots (A to Y) of thylakoidal MPC subunits of *B. juncea* L. obtained from PDQuest analysis of SDS-PAGE gels (2nd dimension) for the four treatments T_0 (control), T_1 (+FS), T_2 (+As) and T_3 (As+FS) at 14 DAT. Arsenic, As; $FeSO_4$, FS.

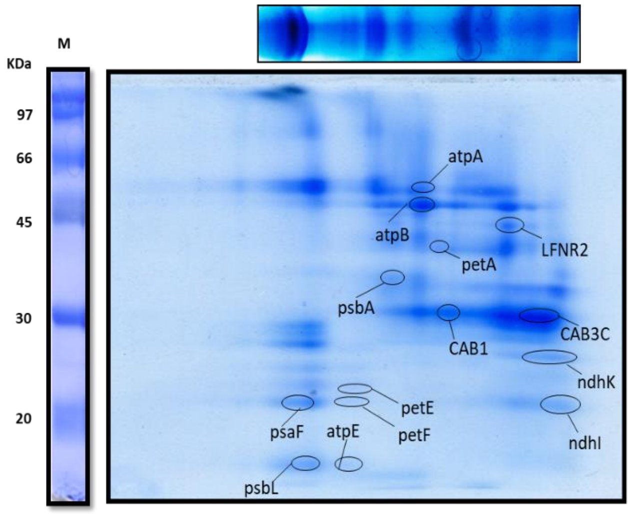

Supplementary Figure S6: Control gel showing differentially expressed subunits of PSI, PSII, ATP synthase, Cyt b6/f, LHCII trimer, LHCII monomer and free proteins.

Supplementary Table S1: Composition of gradient gel for BN-PAGE.

**Gel Components**	**5% gel**	**13% gel**
3X Gel buffer*	2.08 mL	1.66 mL
49% Acrylamide	0.520 mL	1.315 mL
Glycerol	0 mL	0.928 mL
DDW	2.4 mL	1.097 mL
10% APS	28 µL	25 µL
TEMED	2.8 µL	2.5 µL

*3x Gel buffer: 200 mM e-amino-n-caproic acid, 150 mM Bis-Tris, pH 7

Supplementary Table S2: Normalized band volumes of thylakoidal multi-protein-pigment complexes in BN-PAGE gradient gel for the four treatments T_0 (control), T_1 (+$FeSO_4$), T_2 (+As) and T_3 (As+$FeSO_4$). Parenthesis includes percent change over control.

**Band No.**	**T_0 (Control)**	**T_1 (+$FeSO_4$)**	**T_2 (+As)**	**T_3 (As+$FeSO_4$)**
1	636	698 (10)	514 (-19)	683 (7)
2	742	781 (5)	512 (-31)	597 (-20)
3	1060	795 (-25)	461 (-57)	661 (-38)
4	1272	1358 (7)	753 (-41)	1076 (-15)
5	1431	1508 (5)	795 (-44)	1084 (-24)
6	4717	3993 (-15)	1850 (-61)	2361 (-50)
7	7256	7011 (-3)	12365 (70)	11003 (52)
8	5406	3933 (-27)	1678 (-69)	1054 (-81)
9	3339	2766 (-17)	2083 (-38)	2532 (-24)
10	1802	1761 (-2)	3014 (67)	2054 (14)
11	3021	3470 (15)	5254 (74)	4986 (65)

Supplementary Table S3: Identification of bands/thylakoidal membrane protein complexes of
B. juncea L. resolved by BN PAGE (1st dimension). Details in the columns represent Band number along with its molecular weight (kDa), Protein complex name, UniProt ID, Subunit name, Peptide sequence Mr (Da), Peptide sequences, % sequence coverage (% Seq Cov), Gene name, NCBI gene ID and NCBI protein ID for each band identified through MALDI–TOF mass spectrometry.

Band No.	Protein complex name	UniProt ID	Subunit name	Peptide Sequence Mr (Da)	Peptide sequences	% Seq Cov	Gene name	NCBI gene ID	NCBI protein ID
**Band 1**;<br>987 kDa	Supercomplex 1	YCX3_MARPO	Uncharacterized 3.8 kDa protein in ycf12-psaM intergenic region/ ORF30	875.4786<br>1307.6907<br>1117.6324	MELILNK<br>MELILNKEYR<br>YRFFLLLF	60%	ORF30	2702587	NP_039284.1
		YCF4_ANGEV	Photosystem I assembly protein Ycf4	884.508<br>1117.5417<br>1233.6903	IDPIKGSR<br>WGFPGENRR<br>FMIKDIQAIR	14%	ycf4	4788166	YP_001023712.1
**Band 2**;<br>819 kDa	Supercomplex 2		As per Andaluz et al 2006						
**Band 3**;<br>553 kDa	PSI (RCI+LHCI), PSII core dimer, ATP synthase	ATPA_LEMMI	ATP synthase subunit alpha	1002.5345<br>1273.6878<br>1102.6168	ADEISSIIR<br>TAVATDTILNQK<br>VGSAAQIKAMK	6%	atpA	844790	NP_051044
		PSBR_BRACM	Photosystem II 10 kDa polypeptide	1203.6394<br>800.5120	MSARGLPSLTR<br>IVASGVKK	13%	PSBR	103830498	XP_009104547
**Band 4**;<br>382 kDa	PSI core	PSAA_EQUPA	Photosystem I P700 chlorophyll a apoprotein A1 (Fragment)	1067.6339<br>1476.8089<br>1047.5025<br>904.3862	IIVEKDPVR<br>EIPLPHEFILNR<br>YSDFLTFR<br>FPCDGPGR	5%	psaA	844768	NP_051059
		PSAH_HORVU	Photosystem I reaction center subunit VI	1047.5673<br>841.5134	GLSGSSISGRK<br>ARGAAVVAK	13%	PSAH	841653	NP_175633
**Band 5**;<br>355 kDa	Cytochrome b6-f dimer	UCRIA_WHEAT	Cytochrome b6-f complex iron-sulfur subunit	831.4273<br>1492.7715<br>1002.4559	VPDMSKR<br>VVFVPWVETDFR<br>TGDNPWWK	12%	petC	827996	NP_192237
**Band 6**;<br>312 kDa	ATP sythase subunit, PSII core monomer	ATPB_IPOAQ	ATP synthase subunit beta/ ATP synthase CF1 beta subunit	1552.7338<br>1189.6343<br>1044.5968<br>1327.6633<br>1226.5489<br>1432.7674	VRAVAMSDTDGLMR<br>SAPAFIQLDTK<br>VVDLLAPYR<br>AHGGVSVFGGVGER<br>EGNDLYLEMK<br>FVQAGSEVSALLGR	14%	atpB	844757	NP_051066
		PSBD_CHLRE	Photosystem II D2 protein	1278.6932<br>1226.5931<br>1040.5978<br>1552.8613	TIAIGTYQEKR<br>AYDFVSQEIR<br>NILLNEGIR<br>LVFPEEVLPRGNAL	12%	psbD	2716961	NP_958420
**Band 7**;<br>244 kDa	PSII subcomplex	PSBB_CICAR	Photosystem II CP47 reaction center protein	1054.6022<br>1922.8587<br>1484.7300<br>1289.6615<br>1226.6871	TGKPSLDLPK<br>YQWDQGYFQQEIYR<br>LAFYDYIGNNPAK<br>AQLGEIFELDR<br>LGDPSTKKPVVS	11%	psbB	6797495	YP_002149758
		FER_WHEAT	Ferredoxin	831.4636<br>907.5127<br>1040.5502	MAAALSLR<br>AQATYKVK<br>SDIVIETHK	17%	PETF	837639	NP_172565
**Band 8**;<br>168 kDa	LHCII trimer	CB23_POPEU	Chlorophyll a-b binding protein 3/<br>LHCII type I CAB-3	1251.6684<br>981.5243	NRELEVIHSR<br>ELEVIHSR	35%	LHCB1.3/CAB3	839869	NP_564339
		CB21_ARATH	Chlorophyll a-b binding protein 2.1	1630.8103<br>1251.6684<br>981.5243	STPQSIWYGPDRPK<br>NRELEVIHSR<br>ELEVIHSR	9%	LHCB2.1	815058	NP_178585
		CB22_ARATH	Chlorophyll a-b binding protein 2.2	1630.8103<br>1251.6684<br>981.5243	STPQSIWYGPDRPK<br>NRELEVIHSR<br>ELEVIHSR	9%	LHCB2.2	815055	NP_178582
		CB24_ARATH	Chlorophyll a-b binding protein 2.4	1630.8103<br>1251.6684<br>981.5243	STPQSIWYGPDRPK<br>NRELEVIHSR<br>ELEVIHSR	9%	LHCB2.4	822391	NP_189406

Band No.	Protein complex name	UniProt ID	Subunit name	Peptide Sequence Mr (Da)	Peptide sequences	% Seq Cov	Gene name	NCBI gene ID	NCBI protein ID
**Band 9**; 151 kDa	LHCII dimer	CB21_SINAL	Chlorophyll a-b binding protein 1	1276.6412<br>1790.8588<br>1324.6670<br>1054.5229	LSPGASEVFGTGR<br>TVKPTGPSGSPWYGSDR<br>NRELEVIHCR<br>ELEVIHCR	15%	LHCB1.3/ CAB1	839871	NP_174286
		CB5_ARATH	Chlorophyll a-b binding protein CP26 /LHCIIc	1278.6456<br>948.4566<br>1157.6445<br>1246.6458	SSAPLASSPSTFK<br>WYGPDRR<br>IFLPDGLLDR<br>YQAFELIHAR	14%	LHCB5	826626	NP_192772
**Band 10**; 112 kDa	LHCII monomer	CB2B_SOLLC	Chlorophyll a-b binding protein 1B/ LHCII type I CAB-1B	1790.8945<br>1324.6670<br>1054.5229	LSPSASEISGNGRITMR<br>NRELEVIHCR<br>ELEVIHCR	10%	CAB1B	101263969	XP_010316913
		CB2G_SOLLC	Chlorophyll a-b binding protein 3C/ LHCII type I CAB-3C	1790.8945<br>1324.6670<br>1054.5229	LSPSSSEITGNGRVTMR<br>NRELEVIHCR<br>ELEVIHCR	10%	CAB3C	108491835	NP_001316912
		CB5_ARATH	Chlorophyll a-b binding protein CP26 / LHCIIc	1278.6456<br>1157.6445<br>1246.6458	SSAPLASSPSTFK<br>IFLPDGLLDR<br>YQAFELIHAR	11%	LHCB5	826626	NP_192772
**Band 11**; 80 kDa	Free proteins	GLGL3_SOLTU	Glucose-1-phosphate adenylyltransferase large subunit 3	1327.6898<br>1431.6718<br>1708.8454<br>1754.9216<br>880.5130<br>1054.5771	RWFQGTAHAVR<br>MDYLHFVQSHR<br>IDDTGRVMSFSEKPK<br>SFFRANLALTEHPPR<br>IGTNVHLK<br>VPLGIGENTR	14%	AGPS3	102582750	NP_001275395
		FENR2_ORYSJ	Ferredoxin--NADP reductase, leaf isozyme 2	831.4311<br>1431.7466<br>1754.8257<br>1432.6842<br>1044.5491<br>1251.6135	RSSGNGVR<br>ITGPVGKEMLMPK<br>EQTNAAGEKMYIQTR<br>MYIQTRMAEYK<br>DELWELLK<br>DGIDWLDYKK	16%	LFNR2	4328000	XP_015625198
		ICS1_ARATH	Isochorismate synthase 1	1054.5084<br>1246.6306<br>1754.9138<br>1327.5979<br>980.4967	SYSPTPFTR<br>SQPPSFSSGVVR<br>FLSSTSPLIRAYGGMR<br>AYGGMRFDPNGK<br>GAYFPAVEK	9%	ICS1	843810	NP_565090
		NQR2_ORYSJ	Probable NADPH:quinone oxidoreductase 2	1044.5564<br>1157.6516<br>1246.6266	EGSTSPKALR<br>GSANTGLIRAAK<br>AAAIVSASGGSGGSR	18%	Os01g0954000	4325401	XP_015629376

Supplementary Table S4: Identification of subunits of thylakoidal membrane protein complexes of *B. juncea* resolved by SDS PAGE (2nd dimension). Details in the columns represent Spot ID along with its UniProt ID, Homologous protein name, Practical (Pr)/Theoretical (Th) molecular weight (MW) (kDa), Peptide sequence Mr (Da), Peptide sequences, % sequence coverage (% Seq cov), Gene name, NCBI gene ID and NCBI protein ID for each spot identified through MALDI–TOF mass spectrometry.

Spot ID.	UniProt ID	Homologous protein name	Pr/Th MW (KDa)	Peptide Sequence Mr (Da)	Peptide sequences	% Seq Cov	Gene name	NCBI gene ID	NCBI protein ID
**A**	PSAF_ARATH	photosystem I subunit F/ photosystem I subunit III	20-23/24.32	898.4297<br>1224.7078<br>1178.6237	RFDNYGK<br>EIIIDVPLASR<br>GFIWPVAAYR	12	psaF	840021	NP_174418
**B**	PSBL_CHLRE	Photosystem II reaction center protein L	6-9/4.42	1764.9417<br>856.4767	MARPNPNKQVVELNR<br>QVVELNR	39	psbL	2716995	NP_958400.2
**C**	SECA_SPIOL	Protein translocase subunit SecA	97-100/117	860.3876<br>2211.0518<br>1046.5872<br>891.4814<br>1475.7984<br>1273.6812<br>1044.5572<br>1102.5116<br>1318.6663<br>1475.7112<br>876.4593	GGDPAESTK<br>GFNFCVIDEVDSILIDEAR<br>AAKIAAAFER<br>DVNYIIR<br>GKEILIVDEFTGR<br>LGAVTIATNMAGR<br>IREMLMPR<br>SGRQGDPGSSR<br>MLTRALDEAQR<br>KDMVENQAPGLMK<br>DPLIEYK	11	secA	838767	NP_001185059
**D**	ATPE_PINTH	ATP synthase epsilon chain/ ATP CF1 epsilon subunit	7-10/15.03	1141.6455<br>892.4039<br>856.5130	ITILVNNAER<br>EAQENFR<br>IAKADLAR	18	atpE	809050	NP_042414
**E**	PLAS2_POPNI	Plastocyanin B	20-23/17.02	1261.6626<br>1364.6493	AASASNAKVSASAK<br>ISMSEEDLLNAK	15	PETE	103832056	XP_009106261
**F**	FER_CHLRE	Ferredoxin	18-21/13.62	1044.5815	VTLKTPSGDK	7	PETF	5718285	XP_001692808
**G**	TC754_ARATH	Protein TOC75-4	38-41/43.77	2383.192<br>876.4454<br>1319.6656	IPVKNTHVYAFAEHGNDLGSSK<br>GNPTGLYR<br>KMGHGSSYGLGVK	10	TOC75-4	826486	NP_192647
**H**	PAP12_ARATH	Probable plastid-lipid-associated protein 12	47-50/46.07	1081.5556<br>892.5018<br>856.4767	FDRAAFDLK<br>GTTFVLQK<br>ETVPRQK	5	PAP12	841534	NP_175522
**I**	RK5_ARATH	50S ribosomal protein L5	29-32/28.44	1102.5771<br>1096.6029<br>892.4767<br>1081.5669<br>840.3878	FSPLAAPSSAR<br>YVNIHQVPK<br>FDAVGKTR<br>LKSHHFDAK<br>SHHFDAK	14	RPL5	827947	NP_192040
**J**	ATPB_OLTVI	ATP synthase CF1 subunit beta	50-55/51.64	876.4375<br>892.4324<br>1044.5968<br>1200.6979<br>1327.6633<br>1470.7541<br>1432.7674<br>908.5080	MNVSVEAK<br>MNVSVEAK<br>VVDLLAPYR<br>VVDLLAPYRR<br>AHGGVSVFGGVGER<br>VGLTALTMAEYFR<br>FVQAGSEVSALLGR<br>TTLQRYK	13	atpB	4100137	YP_635877.1
**K**	ATPA_POPAL	ATP synthase subunit alpha	56-60/55.34	892.425<br>875.4899<br>1552.7310<br>1102.6168	GAISSSESR<br>QMSLLLR<br>EAYPGDVFYLHSR<br>VGSAAQIKAMK	7	atbA	4178218	YP_665543.1
**L**	YCF4_CHLAT	Photosystem I assembly protein Ycf4	27-30/21.51	1102.6247<br>840.4566<br>875.5844	QDGIVRIFR<br>EGINPRR<br>VIYLKIK	12	ycf4	124112130	YP_001019142.1
**M**	PSBA_CHLRE	Photosystem II protein D1/ photosystem II P680 reaction center D1 protein	34-37/39.24	1117.5628<br>1458.7255<br>1313.7092	RENSSLWAR<br>LIFQYASFNNSR<br>VLNTWADIINR	9	psbA	2716969	NP_958377

Spot ID.	UniProt ID	Homologous protein name	Pr/Th MW (KDa)	Peptide Sequence Mr (Da)	Peptide sequences	% Seq Cov	Gene name	NCBI gene ID	NCBI protein ID
**N**	PORB_ARATH	Protochlorophyllide reductase B /NADPH-protochlorophyllide oxidoreductase B	39-42/43.56	924.4454<br>910.5025<br>946.5276	QFVDNFR<br>EHIPLFR<br>ALFPPFQK	5	PORB	828853	NP_001031731
**O**	DJ1B_ARATH	Protein DJ-1 homolog B	40-44/47.47	908.4617<br>856.5243<br>860.5153	LHHYSPR<br>ISLRVNR<br>LVNMLKK	4	DJ1B	841762	NP_564626
**P**	CB21_SINAL	Chlorophyll a-b binding protein 1/ LHCII type I CAB-1	28-31/28.32	1706.9025<br>1790.8588<br>1324.6670<br>1054.5229	AASTMALSSPAFAGKAVK<br>TVKPTGPSGSPWYGSDR<br>NRELEVIHCR<br>ELEVIHCR	16	CAB1	839871	NP_174286
**Q**	CYF_MAIZE	Cytochrome f	37-40/35.60	908.512<br>1006.5157<br>856.5130	KTFSWLK<br>IPYDMQLK<br>QVLANGKK	7	petA	845191	NP_043037
**R**	ATPB_OLTVI	ATP synthase subunit beta	50-54/51.64	876.4375<br>892.4324<br>1044.5968<br>1200.6979<br>1470.8479<br>1327.6633<br>1432.7674<br>908.5080	MNVSVEAK<br>MNVSVEAK<br>VVDLLAPYR<br>VVDLLAPYRR<br>TVLIMELINNIAK<br>AHGGVSVFGGVGER<br>FVQAGSEVSALLGR<br>TTLQRYK	13	atpB	4100137	YP_635877.1
**S**	ATPA_NASOF	ATP synthase subunit alpha	56-60/54.91	1106.5468<br>1415.7772<br>1251.7187<br>1473.7787<br>1552.7310<br>902.5702	ERIEQYNR<br>IAQIPVSEAYLGR<br>LIESPAPGIISR<br>ASSVAQVVTSLQER<br>EAYPGDVFYLHSR<br>KFLVQLR	13	atbA	4962216	YP_001123799.1
**T**	CYF_CARPA	Cytochrome f	33-36/35.3	860.4756<br>856.5130<br>1037.5294	NTLSWIK<br>QVLANGKK<br>YPIYVGGNR	7	petA	5878447	YP_001671696
**U**	CB2G_SOLLC	Chlorophyll a-b binding protein 3C/ LHCII type I CAB-3C	28-31/28.47	1474.6974<br>1324.6670<br>1054.5229	ATSTMALSSSTFAGK<br>NRELEVIHCR<br>ELEVIHCR	9	CAB3C	108491835	NP_001316912
**V**	FNRL2_ARATH	Ferredoxin--NADP reductase, leaf isozyme 2	42-45/41.48	2383.1941<br>2705.3591<br>1708.8858<br>1474.6949	LYSIASSALGDLGNSETVSLCVK<br>EMLMPKDPNATVIMLATGTGIAPF<br>R MAQYAAELWELLKK<br>KDNTFVYMCGLK	19	LFNR2	838591	NP_001077566
**W**	NDHI_PIPCE	NAD(P)H-quinone oxidoreductase subunit I	18-21/21.55	1706.874<br>1306.6438<br>1178.5489	YIGQSFAITLSHTNR<br>KMTDKPLDSEK<br>MTDKPLDSEK	14	ndhI	4363701	YP_784524.1
**X**	NDHK_CERDE	NAD(P)H-quinone oxidoreductase subunit K	24-27/26.09	1918.0193<br>1474.8065	SSPRQADLILTAGTITMK<br>QADLILTAGTITMK	7	ndhK	5729382	YP_001542451.1
**Y**	EAAC_ARATH	Probable envelope ADP, ATP carrier protein	38-41/41.97	856.4919<br>1065.5529<br>892.4290	AILTFHR<br>FACISLVEK<br>SLPEEYR	6	EAAC	824350	NP_190755
